# Barriers to healthcare access in patients with chronic pain or potential migraine in Japan: a cross-sectional internet survey

**DOI:** 10.3389/fpain.2023.1271438

**Published:** 2023-10-03

**Authors:** Yusuke Karasawa, Keisuke Yamaguchi, Shogo Nakano, Kazutaka Nozawa, Masako Iseki

**Affiliations:** ^1^ Department of Pain Medicine, Juntendo University Graduate School of Medicine, Bunkyo-ku, Tokyo, Japan; ^2^ Medical Affairs, Viatris Pharmaceuticals Japan Inc., Minato-ku, Tokyo, Japan

**Keywords:** authorized generic, chronic pain, economic burden, generic drug, migraine, patient burden

## Abstract

**Purpose:**

Chronic pain and migraines often go untreated despite patient- and economic-related burdens (e.g., impaired quality of life and productivity). Understanding the reasons for non-treatment is important to enable interventions aimed at improving care-seeking behaviors. However, reports on disease-specific justifications for nontreatment in Japan are limited. We aimed to determine the barriers to healthcare access in untreated patients with chronic pain or migraines.

**Patients and methods:**

This was a non-interventional, cross-sectional, internet questionnaire survey of patients with chronic pain or migraines. The primary endpoint was to identify the reasons for untreated chronic pain or migraines. Secondary endpoints included factors associated with healthcare access, including patient background, patient-reported outcomes, and awareness of generic or authorized generic drugs (AG).

**Results:**

We surveyed 1,089 patients with chronic pain [605 (55.6%) untreated] and 932 patients with migraines [695 (74.6%) untreated] in 2021. The main reasons for not seeking treatment for chronic pain was “my pain is tolerable” and for migraine, “I can manage my pain with over-the-counter drugs.” Background factors significantly associated with untreated chronic pain were younger age, less time required to access the nearest medical institution, less pain, higher activities of daily living (ADL) scores, and lower awareness of generic drugs and AG. Among patients with migraine, notable characteristics included being female, having shorter travel times to the nearest medical facility, residing in municipalities with populations under 50,000, experiencing moderate to severe pain, having higher ADL scores, and displaying lower awareness of AG. The AG awareness rate was 2-fold higher in treated patients than in untreated patients.

**Conclusion:**

Educating patients regarding the risks associated with pain and its underlying causes, availability of inexpensive treatment options, and location of appropriate treatment facilities may increase treatment rates.

## 1. Introduction

Noncommunicable diseases (NCDs), such as cardiovascular disease, cancer, and chronic respiratory disease, are associated with high mortality rates and reduced quality of life (QOL) (1). Pain is one of the most common symptoms for many NCDs, especially those related to musculoskeletal diseases or migraine headaches (2, 3). Chronic pain and migraine are representative of diseases that cause pain, and both are known to greatly impact social and daily activities and lower QOL (4–6).

In Japan in 2013, the prevalence of chronic pain was 16.6% according to a survey among 10,000 randomly selected individuals aged ≥20 years (7). The overall work loss due to chronic pain is estimated to be ¥1953 billion (US$19.9 billion) per year (8). Treatments for chronic pain also greatly impact health economics because they tend to be expensive and require prolonged treatment schedules (9).

Migraine is associated with symptoms such as sensitivity to light, sound, and nausea (10). The most recent study of 21,480 people in Japanese health insurance societies from December 1, 2017, to November 30, 2020, reported a migraine prevalence of 3.2% (11). Patients with migraine experience decreased productivity (11), which has been identified as the main cause of years lived with disability in the population aged 15–49 years (12).

Although pain may be a common reason for seeking medical treatment, more than half of individuals with preexisting pain remain untreated (chronic pain:55% (9); migraine:69.4% (13)). Appropriate treatment interventions may reduce patient burden by improving QOL and activities of daily living (ADL) and reduce the economic burden caused by decreased productivity in patients with chronic pain (8) or migraines (14). Furthermore, pain may arise from an as-yet undiagnosed disease that can be detected and treated through medical consultations. Therefore, improved healthcare access for patients with chronic pain or migraines is urgently required. Health care barriers are caused by financial, structural, and cognitive-related factors (15). Regarding financial factors, the influence of inexpensive treatment options such as generic drugs or authorized generic (AG), which have been garnering increased awareness nowadays, on care-seeking behavior is unknown. As a starting point, understanding the real-world situation and reasons for such barriers to treatment would likely inform interventions aimed at improving care-seeking behaviors among those who need it. However, data on disease-specific justifications for non-treatment in Japan are limited.

Therefore, the current study was conducted to determine the reasons why patients with chronic pain or migraines might not seek treatment and to reveal the factors associated with healthcare access barriers.

## 2. Materials and methods

### 2.1. Study design

This was a non-interventional, cross-sectional, internet questionnaire survey of Japanese patients with chronic pain or migraines. Data were collected in October 2021 by Cross Marketing Inc. (Shinjuku-ku, Tokyo, Japan, hereafter referred to as the survey agency), who administered a large general population questionnaire panel in Japan, and coding was conducted by QLife Inc. (Minato-ku, Tokyo, Japan). Prior to the main study, a preliminary survey was conducted to determine the reasons for untreated chronic pain or migraines. The responses to the open-ended questions obtained in the pre-survey were collated using QLife and used as the closed-ended answer choices in the main survey.

The questionnaires in the main survey were distributed on the Cross Marketing internet site under the heading “Questionnaire about you,” so that the content of the questionnaire could not be identified from the title. People who accessed the site then read an explanatory document about the study (stating that the survey was about chronic pain and migraines and that questions would be asked to ascertain pain and patient status). Those who decided to participate in the study then clicked on the “I agree (to participate in the study)” button to provide informed consent and proceed. Participants answered screening questions to determine their eligibility for the study. Those who fulfilled the eligibility criteria responded to a follow-up questionnaire, and the survey agency collected participants' information from the forms.

To improve the generalizability of the results of this study, both the pre-survey and the main survey were designed based on the results of the 2020 Japanese national census (16). Questionnaires were distributed so that sex, age, and geographic location or participants represented the overall demographics of Japan. The target number of participants in the preliminary survey was 100 untreated patients with chronic pain and 100 untreated patients with migraines.

The study protocol was approved by the Takahashi Clinic Ethics Committee, a third-party ethics review committee unaffiliated with Viatris Pharmaceuticals. The study was conducted in compliance with the legal and regulatory requirements and the Ethical Guidelines for Medical and Health Research Involving Human Subjects established by the Ministry of Health, Labour and Welfare and the Ministry of Education, Culture, Sports, Science and Technology. All the study participants provided informed consent.

### 2.2. Participants

The inclusion criteria for patients with chronic pain were: aged ≥20 years with chronic pain that had persisted for ≥3 months, had been present within the last month, and was rated 5–10 on the pain Numerical Rating Scale (NRS). “Treated” or “untreated” was determined by whether or not the patient had received any kind of treatment for chronic pain in the past year. Patients were excluded if their pain was caused by migraines or cancer.

The inclusion criteria for patients with potential migraine were: aged ≥20 years who answered “sometimes” or “more than half the time” to at least two items on the simple migraine questionnaire (17). “Treated” or “untreated” was determined by whether or not the patient had received any kind of treatment for migraine in the past year. There were no exclusion criteria for patients with migraines.

### 2.3. Data collection

The questionnaires used in this study are shown in Supplementary Tables S1–S4, in Supplemental Digital Content 1. During the preliminary survey, data regarding the reasons for untreated chronic pain or migraines were collected. The main survey collected data such as sex, age, occupation, education, area of residence (municipality with a population <50,000, 50,000–100,000, or ≥100,000), time required to reach the nearest treatment setting, past hospital visits (for chronic pain or migraine), household income, pain NRS, duration of pain, details of consultations at medical facilities or other settings [if yes, reason for previous treatment; if none, treatment at non-medical facilities (use of over-the-counter drugs), reason for not receiving treatment, and factors that may change the person's intention to visit a treatment setting], awareness of generic drugs, awareness of authorized generic drugs (AG), health literacy (European Health Literacy Survey Questionnaire) (18), QOL [EuroQol 5 dimensions 5-level (EQ-5D-5l)] (19), ADL [Pain Disability Assessment Scale (PDAS)] (20), and labor productivity [work productivity and activity impairment (WPAI)] (21). In addition, the PainDETECT (22) and Migraine Disability Assessment Scale (23, 24) were administered to patients with chronic pain and migraines, respectively. The evaluation methods for the questionnaires used in this survey are summarized in the Supplemental Methods, Supplemental Digital Content 1.

### 2.4. Endpoints

The primary endpoint was the reason for not seeking treatment in untreated patients with chronic pain or migraines. The selection and order of answers were decided based on the preliminary survey outcomes to mitigate bias prior to the implementation of the main survey. Secondary endpoints included factors associated with barriers to healthcare access, reasons for healthcare visits in treated patients, and awareness of generic drugs/AG between treated and untreated patients.

### 2.5. Statistical analysis

For demographic and clinical characteristics, data were summarized using descriptive statistics, including mean ± standard deviation (SD), median, quartile [Q]1, and Q3, for continuous variables and *n* (%) for categorical variables. For patients with untreated chronic pain or migraines, the reasons for remaining untreated were summarized using descriptive statistics, and the background factors associated with nontreatment were evaluated using logistic regression analysis. For the two variables, with Spearman's correlation coefficient of 0.4 or higher in the univariate analysis, logistic regression analysis was conducted, leaving only the representative variable and excluding the rest. The potential impact of awareness of generic drugs and AG on healthcare visits was also summarized using descriptive statistics.

The stratification by age (young [20–39 years], middle-age [40–64 years], and older patients [≥65 years]) used in the univariate and multivariate analysis was based on the definitions from the “Act on Promotion of Development and Support for Children and Young People,” the Cabinet Office Survey, and the World Health Organization (25–27). All statistical analyses were conducted using the SAS software (version 9.4; SAS Institute Inc., Cary, NC, USA).

## 3. Results

### 3.1. Patient background

#### 3.1.1. Chronic pain

**Figure 1:**
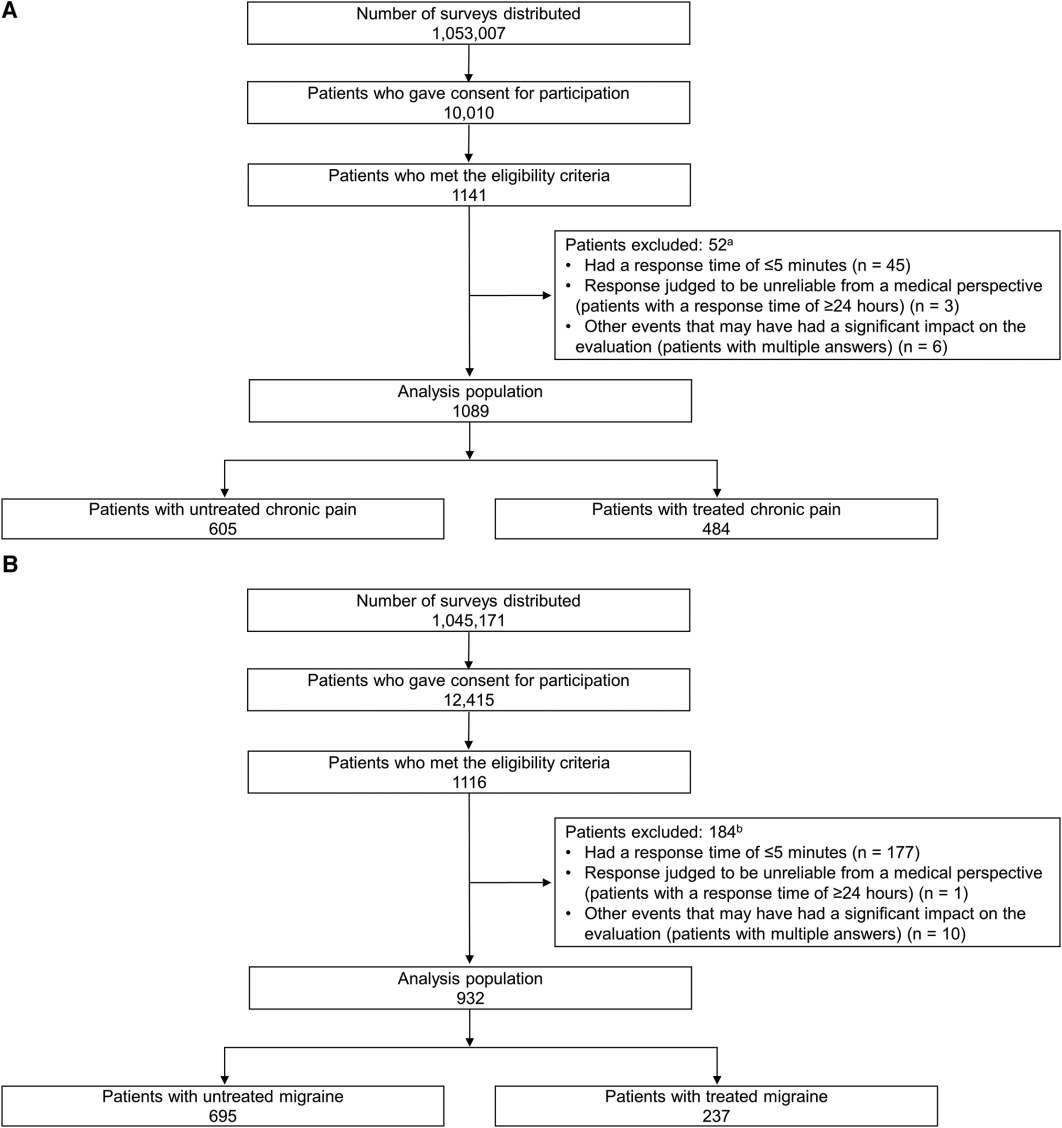
Flow chart of patients with (**A**) chronic pain and (**B**) migraine. ^a^The total number was 52 because two patients met both of the following criteria: “Participants with a response time of ≥24 h” and “Participants with multiple answers.” ^b^The total number was 184 because four patients met both of the following criteria: “Participants with a response time of ≤5 min” and “Participants with multiple answers.”.

A flowchart of patients with chronic pain is shown in Figure 1A. Of the 1,053,007 surveys distributed, 10,010 patients (1.0%) consented to participate in the study, and 1,141 (11.4%) met the eligibility criteria. In total, 52 patients were excluded because they did not fulfill the analysis criteria, leaving 1,089 patients in the analysis population (605 [55.6%] untreated and 484 [44.4%] treated). The characteristics of the untreated and treated patients are as follows: male, 50.6% and 53.9%; mean ± SD pain NRS scores, 6.2 ± 1.1 and 6.6 ± 1.2; mean ± SD absenteeism scores, 7.9% ± 20.6% and 9.7% ± 23.3%; mean ± SD costs lost based on absenteeism, 495,821.4 ± 1,794,738.9 ¥/year and 641,328.7 ± 1,926,706.3 ¥/year; mean ± SD presenteeism scores, 35.3% ± 26.8% and 40.7% ± 26.3%; mean ± SD costs lost based on presenteeism, 1,312,850.1 ± 1,069,630.5 ¥/year and 1,559,728.7 ± 1,107,228.9 ¥/year; proportions of patients with PainDETECT scores <12, 75.2% and 62.8%; and mean ± SD durations of pain, 78.3 ± 97.5 and 79.0 ± 99.3 months, respectively (Table 1).

**Table 1:** Patient demographic and clinical characteristics.

	Chronic Pain		Potential Migraine	
	Untreated *n* = 605	Treated *n* = 484	Untreated *n* = 695	Treated *n* = 237
Sex (male), *n* (%)	306 (50.6)	261 (53.9)	250 (36.0)	122 (51.5)
Age, years				
Mean ± SD	50.0 ± 13.7	53.4 ± 14.3	41.7 ± 12.6	42.8 ± 13.4
20–39, *n* (%)	148 (24.5)	91 (18.8)	328 (47.2)	116 (48.9)
40–64, *n* (%)	362 (59.8)	281 (58.1)	339 (48.8)	106 (44.7)
≥65, *n* (%)	95 (15.7)	112 (23.1)	28 (4.0)	15 (6.3)
Occupation, *n* (%)				
Manual labor	92 (15.2)	73 (15.1)	110 (15.8)	47 (19.8)
Customer service, sales	114 (18.8)	72 (14.9)	160 (23.0)	61 (25.7)
Office work	207 (34.2)	174 (36.0)	209 (30.1)	85 (35.9)
Student	4 (0.7)	4 (0.8)	16 (2.3)	2 (0.8)
Unemployed	188 (31.1)	161 (33.3)	200 (28.8)	42 (17.7)
Education, *n* (%)				
Junior high school/high school/vocational	269 (44.5)	196 (40.5)	319 (45.9)	91 (38.4)
Junior college/university	311 (51.4)	259 (53.5)	327 (47.1)	129 (54.4)
Graduate school	25 (4.1)	29 (6.0)	49 (7.1)	17 (7.2)
Population of municipality of residence, *n* (%)				
≥100,000	399 (66.0)	342 (70.7)	401 (57.7)	150 (63.3)
50,000–100,000	124 (20.5)	86 (17.8)	164 (23.6)	65 (27.4)
<50,000	82 (13.6)	56 (11.6)	130 (18.7)	22 (9.3)
Access to a healthcare institution				
Time required to access the nearest medical institution, min				
Median (Q1, Q3)	10.0 (5.0, 15.0)	15.0 (10.0, 27.5)	10.0 (5.0, 15.0)	15.0 (10.0, 30.0)
Past healthcare institution visits (history of visits due to chronic pain or migraine), *n* (%)				
Medical facility				
Yes	0 (0.0)	375 (77.5)	0 (0.0)	213 (89.9)
None	605 (100.0)	109 (22.5)	695 (100.0)	24 (10.1)
Complementary therapy facility				
Yes	0 (0.0)	160 (33.1)	0 (0.0)	80 (33.8)
None	605 (100.0)	324 (66.9)	695 (100.0)	157 (66.2)
Household income (¥ [US$]), *n* (%)^a^				
<1 million [<9,091]	43 (7.1)	40 (8.3)	93 (13.4)	27 (11.4)
≥1 to <2 million [≥9,091 to <18,182]	53 (8.8)	34 (7.0)	71 (10.2)	22 (9.3)
≥2 to <3 million [≥18,182 to <27,273]	59 (9.8)	65 (13.4)	91 (13.1)	27 (11.4)
≥3 to <4 million [≥27,273 to <36,364]	79 (13.1)	61 (12.6)	83 (11.9)	30 (12.7)
≥4 to <5 million [≥36,364 to <45,455]	84 (13.9)	64 (13.2)	92 (13.2)	22 (9.3)
≥5 to <6 million [≥45,455 to <54,545]	78 (12.9)	46 (9.5)	59 (8.5)	22 (9.3)
≥6 to <7 million [≥54,545 to <63,636]	45 (7.4)	42 (8.7)	41 (5.9)	14 (5.9)
≥7 to <8 million [≥63,636 to <72,727]	35 (5.8)	41 (8.5)	47 (6.8)	21 (8.9)
≥8 to <10 million [≥72,727 to <90,909]	63 (10.4)	41 (8.5)	52 (7.5)	16 (6.8)
≥10 million [≥90,909]	66 (10.9)	50 (10.3)	66 (9.5)	36 (15.2)
Chronic pain status				
NRS				
Mean ± SD	6.2 ± 1.1	6.6 ± 1.2	4.0 ± 2.6	5.1 ± 2.6
Low, *n* (%)	0 (0.0)	0 (0.0)	388 (55.8)	98 (41.4)
Moderate, *n* (%)	526 (86.9)	374 (77.3)	237 (34.1)	100 (42.2)
Severe, *n* (%)	79 (13.1)	110 (22.7)	70 (10.1)	39 (16.5)
Duration, months				
Mean ± SD	78.3 ± 97.5	79.0 ± 99.3	109.9 ± 131.3	119.1 ± 126.7
Healthcare consultation (none), *n* (%)				
Consultation at non-medical facilities				
Complementary therapy	0 (0.0)	160 (33.1)	0 (0.0)	80 (33.8)
Over-the-counter drugs	247 (40.8)	0 (0.0)	410 (59.0)	0 (0.0)
Other	0 (0.0)	11 (2.3)	0 (0.0)	7 (3.0)
Factors that may change the person's intention to receive a healthcare consultation				
Short waiting time	133 (22.0)		158 (22.7)	
Healthcare institution located nearby	50 (8.3)		70 (10.1)	
Assured high quality treatment at minimum cost	240 (39.7)		235 (33.8)	
Healthcare professional's kindness during consultation	87 (14.4)		125 (18.0)	
Other	95 (15.7)		107 (15.4)	
Awareness of generic drugs, Yes, *n* (%)	579 (95.7)	475 (98.1)	591 (85.0)	216 (91.1)
Awareness of AG drugs, Yes, *n* (%)	49 (8.1)	74 (15.3)	99 (14.2)	85 (35.9)
Health literacy (HLS-EU-Q47), *n* (%)				
0–25 (insufficient)	227 (37.5)	173 (35.7)	260 (37.4)	86 (36.3)
25–33 (problematic)	156 (25.8)	148 (30.6)	188 (27.1)	66 (27.8)
33–42 (sufficient)	95 (15.7)	66 (13.6)	83 (11.9)	41 (17.3)
42–50 (excellent)	39 (6.4)	45 (9.3)	41 (5.9)	18 (7.6)
Quality of life (EQ-5D-5l)				
Utility index				
Mean ± SD	0.7 ± 0.1	0.7 ± 0.1	0.8 ± 0.2	0.7 ± 0.2
VAS				
Mean ± SD	61.6 ± 19.4	56.6 ± 20.6	64.8 ± 20.7	60.7 ± 21.8
Activities of daily living (PDAS), *n* (%)				
<10	433 (71.6)	256 (52.9)	446 (64.2)	102 (43.0)
≥10	172 (28.4)	228 (47.1)	249 (35.8)	135 (57.0)
Labor productivity, work productivity, and activity impairment				
Absenteeism^b^ (%)				
*N*	365	276	414	151
Mean ± SD	7.9 ± 20.6	9.7 ± 23.3	10.7 ± 23.5	14.0 ± 23.8
Median (Q1, Q3)	0.0 (0.0, 0.0)	0.0 (0.0, 3.8)	0.0 (0.0, 6.3)	0.0 (0.0, 17.4)
Cost lost (¥/year)^c^				
Mean ± SD	495,821.4 ± 1,794,738.9	641,328.7 ± 1,926,706.3	425,116.4 ± 1,451,077.2	693,049.2 ± 1,755,098.2
Median (Q1, Q3)	0.0 (0.0, 0.0)	0.0 (0.0, 184,934.0)	0.0 (0.0, 172,363.0)	0.0 (0.0, 677,265.1)
Cost lost (US$/year)				
Mean ± SD	4,507.5 ± 16,315.8	5,830.3 ± 17,515.5	3,864.7 ± 13,191.6	6,300.4 ± 15,955.4
Median (Q1, Q3)	0.0 (0.0, 0.0)	0.0 (0.0, 1,681.2.0)	0.0 (0.0, 1,566.9)	0.0 (0.0, 6,157.0)
Presenteeism^d^(%)				
*N*	359	268	401	148
Mean ± SD	35.3 ± 26.8	40.7 ± 26.3	39.6 ± 25.3	46.6 ± 23.4
Median (Q1, Q3)	30.0 (10.0, 60.0)	50.0 (20.0, 60.0)	40.0 (20.0, 60.0)	50.0 (30.0, 60.0)
Cost lost (¥/year)^c^				
Mean ± SD	1,312,850.1 ± 1,069,630.5	1,559,728.7 ± 1,107,228.9	1,371,644.6 ± 917,193.1	1,728,604.1 ± 957,203.1
Median (Q1, Q3)	1,184,640.0 (347,040.0, 1,980,000.0)	1,557,000.0 (574,560.0, 2,277,240.0)	1,480,800.0 (620,400.0, 1,969,800.0)	1,767,120.0 (988,920.0, 2,396,520.0)
Cost lost (US$/year)^a^				
Mean ± SD	11,935.0 ± 9,723.9	14,179.4 ± 10,065.7	12,469.5 ± 8,338.1	15,714.6 ± 8,701.8
Median (Q1, Q3)	10,769.5 (3,154.9, 18,000.0)	14,154.5 (5,223.3, 20,702.2)	13,461.8 (5,640.0, 17,907.3)	16,064.7 (8,990.2, 21,786.5)
Total work productivity impairment (%)				
*N*	359	268	401	148
Mean ± SD	38.7 ± 28.4	43.5 ± 27.9	43.2 ± 27.1	52.0 ± 26.2
Median (Q1, Q3)	40.0 (10.0, 60.0)	50.0 (20.0, 63.0)	50.0 (20.0, 61.9)	60.0 (30.0, 70.2)
Activity impairment (%)				
*N*	605	484	695	237
Mean ± SD	38.6 ± 26.8	45.6 ± 27.9	41.3 ± 25.9	48.7 ± 24.3
Median (Q1, Q3)	40.0 (20.0, 60.0)	50.0 (20.0, 70.0)	50.0 (20.0, 60.0)	50.0 (30.0, 70.0)
PainDETECT^e^, *n* (%)				
<12	455 (75.2)	304 (62.8)		
12–19	124 (20.5)	130 (26.9)		
≥19	26 (4.3)	50 (10.3)		
VAS^f^				
Mean ± SD			3.8 ± 2.9	4.6 ± 3.0
Migraine Disability Assessment Scale (Japanese version)^f^, *n* (%)				
Little or no disability in daily life			463 (66.6)	131 (55.3)
Mild disability in daily life			79 (11.4)	28 (11.8)
Moderate disability in daily life			71 (10.2)	40 (16.9)
Severe disability in daily life			82 (11.8)	38 (16.0)

^a^ Calculated using the average 2,021 exchange rate of ¥110 = USD$1.

^b^ Absence due to sickness.

^c^ Calculated using the mean hourly labor fee of the Japanese workforce.

^d^ Decreased labor performance of workers present at work due to health problems.

^e^ Only for the chronic pain survey.

^f^ Only for the migraine survey.

AG, authorized generic drugs; EQ-5D-5l, EuroQol 5 dimensions 5-level; HLS-EU-Q47, European health literacy survey questionnaire; NRS, numerical rating scale; PDAS, pain disability assessment scale; Q, quartile; SD, standard deviation; VAS, visual analog scale.

#### 3.1.2. Potential migraine

A flowchart of the patients with migraines is shown in Figure 1B. Of the 1,045,171 surveys distributed, 12,415 patients (1.2%) consented to participate in the study, and 1,116 (9.0%) met the eligibility criteria. A total of 184 patients were excluded because they did not fulfil the analysis criteria, and 932 patients were included in the analysis (695 [74.6%] untreated and 237 [25.4%] treated). The characteristics of the untreated and treated patients are as follows: male, 36.0% and 51.5%; mean ± SD ages, 41.7 ± 12.6 and 42.8 ± 13.4 years; mean ± SD pain NRS scores, 4.0 ± 2.6 and 5.1 ± 2.6; mean ± SD absenteeism scores, 10.7% ± 23.5% and 14.0% ± 23.8%; mean ± SD costs lost based on absenteeism, 425,116.4 ± 1,451,077.2 ¥/year and 693,049.2 ± 1,755,098.2 ¥/year; mean ± SD presenteeism scores, 39.6% ± 25.3% and 46.6% ± 23.4%; mean ± SD costs lost based on presenteeism, 1,371,644.6 ± 917,193.1 ¥/year and 1,728,604.1 ± 957,203.1 ¥/year; proportions with Migraine Disability Assessment Scale scores showing little or no disability in daily life, 66.6% and 55.3%; and mean ± SD durations of pain, 109.9 ± 131.3 and 119.1 ± 126.7 months, respectively (Table 1).

### 3.2. Primary endpoint

#### 3.2.1. Chronic pain

**Figure 2:**
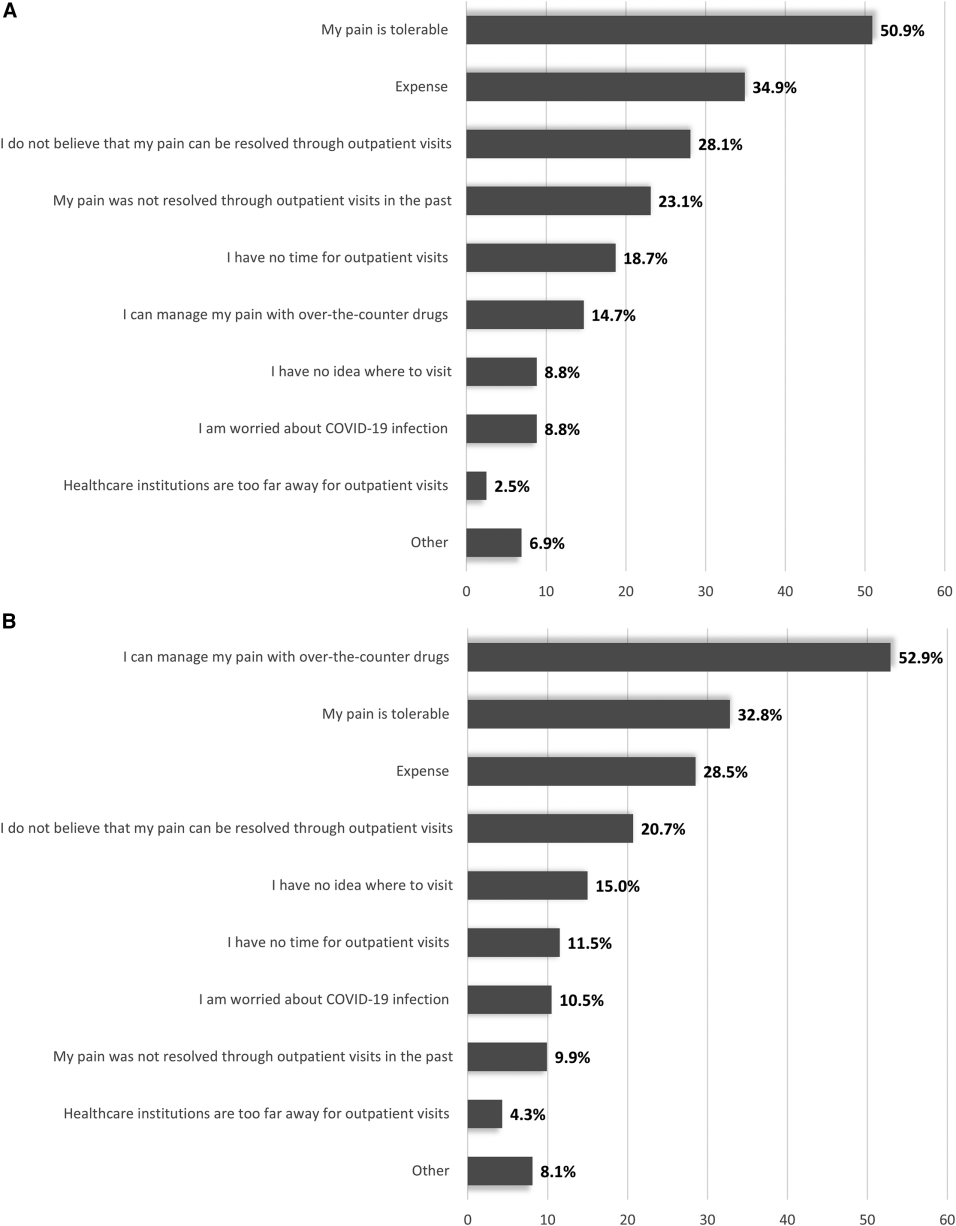
Reasons for not seeking treatment in untreated patients with (**A**) chronic pain and (**B**) migraines (primary endpoint)^a^ More than one reason allowed.

The most common reason for patients with chronic pain not seeking treatment was “my pain is tolerable” (50.9% when multiple reasons were allowed and 34.5% when only one reason was allowed) (see Figure 2A and Supplementary Figure 1A, Supplemental Digital Content 2). The second and third most common reasons for not seeking treatment were “expense” (34.9%) and “I do not believe that my pain can be resolved through outpatient visits” (28.1%), when multiple reasons were allowed, and “expense” (16.7%) and “I do not believe that my pain can be resolved through outpatient visits” (14.2%) when only one reason was allowed.

#### 3.2.2. Potential migraine

The most common reason for patients with migraine not seeking treatment was “I can manage my pain with over-the-counter drugs” (52.9% when multiple reasons were allowed and 40.3% when only one reason was allowed) (see Figure 2B and Supplementary Figure 1B, Supplemental Digital Content 2). The second and third most common reasons for not seeking treatment were “my pain is tolerable” (32.8%) and “expense” (28.5%), when multiple reasons were allowed, and “my pain is tolerable” (13.4%) and “expense” (13.2%) when only one reason was allowed.

### 3.3. Secondary endpoints

#### 3.3.1. Chronic pain

The factors associated with the non-treatment of patients with chronic pain by univariate and multivariate logistic regression analyses are summarized in Table 2. By multivariate logistic regression analysis, background factors significantly associated with access barriers were younger age (age 40–64 years, odds ratio [OR]: 0.8; 95% confidence interval [CI]: 0.6–1.1; age ≥65 years, OR: 0.5; 95% CI: 0.3–0.7), less time required to access the nearest medical institution (OR: 0.7; 95% CI: 0.7–0.8), moderate pain (severe, OR: 0.7; 95% CI: 0.5–1.0), higher ADL (PDAS score ≥10, OR: 0.6; 95% CI: 0.5–0.8), and lower awareness rate of generic drugs (OR: 2.4; 95% CI: 1.1–5.3), and AG (OR: 2.0; 95% CI: 1.3–3.0).

**Table 2:** Factors associated with non-treatment in patients with chronic pain by univariate and multivariate logistic regression analysis.

	Total number of patients	Univariate		Multivariate	
		Odds ratio estimate^a^ (95% CI)	*p* value	Odds ratio estimate^a^ (95% CI)	*p* value
Sex			0.272		
Male (reference)	567				
Female	522	1.1 (0.9–1.5)			
Age (years)			0.003		0.002
20–39 (reference)	239				
40–64	643	0.8 (0.6–1.1)		0.8 (0.6–1.1)	
≥65	207	0.5 (0.4–0.8)		0.5 (0.3–0.7)	
Occupation			0.523		
Manual labor (reference)	165				
Customer service, sales	186	1.3 (0.8–1.9)			
Office work	381	0.9 (0.7–1.4)			
Student	8	0.8 (0.2–3.3)			
Unemployed	349	0.9 (0.6–1.3)			
Education			0.213		
Junior high school/high school/vocational (reference)	465				
Junior college/university	570	0.9 (0.7–1.1)			
Graduate school	54	0.6 (0.4–1.1)			
Access to a healthcare institution			<0.001		<0.001
Time required to access the nearest medical institution (min) (unit: 10 min.)	1,089	0.7 (0.6–0.8)		0.7 (0.7–0.8)	
Population of municipality of residence			0.253		
≥100,000 (reference)	741				
50,000–100,000	210	1.2 (0.9–1.7)			
<50,000	138	1.3 (0.9–1.8)			
Household income [¥ (US$)], *n* (%)^b^			0.215		
<1 million [<9,091] (reference)	83				
≥1 to <2 million [≥9,091 to <18,182]	87	1.5 (0.8–2.7)			
≥2 to <3 million [≥18,182 to <27,273]	124	0.8 (0.5–1.5)			
≥3 to <4 million [≥27,273 to <36,364]	140	1.2 (0.7–2.1)			
≥4 to <5 million [≥36,364 to <45,455]	148	1.2 (0.7–2.1)			
≥5 to <6 million [≥45,455 to <54,545]	124	1.6 (0.9–2.8)			
≥6 to <7 million [≥54,545 to <63,636]	87	1.0 (0.5–1.8)			
≥7 to <8 million [≥63,636 to <72,727]	76	0.8 (0.4–1.5)			
≥8 to <10 million [≥72,727 to <90,909]	104	1.4 (0.8–2.6)			
≥10 million [≥90,909]	116	1.2 (0.7–2.2)			
Chronic pain status (NRS)			<0.001		0.036
Moderate (reference)	900				
Severe	189	0.5 (0.4–0.7)		0.7 (0.5–1.0)	
Duration (months)	1,089	1.0 (1.0–1.0)	0.904		
Health literacy (HLS-EU-Q47)			0.134		
0–25 (insufficient) (reference)	400				
25–33 (problematic)	304	0.8 (0.6–1.1)			
33–42 (sufficient)	161	1.1 (0.8–1.6)			
42–50 (excellent)	84	0.7 (0.4–1.1)			
Quality of life (EQ-5D-5l)					
Utility index (unit: 0.1)	1,089	1.4 (1.2–1.5)	<0.001		
VAS (unit: 10)	1,089	1.1 (1.1–1.2)	<0.001		
Activities of daily living (PDAS)			<0.001		<0.001
<10 (reference)	689				
≥10	400	0.4 (0.3–0.6)		0.6 (0.5–0.8)	
Awareness of generic drugs			0.023		0.033
Yes (reference)	1,054				
No	35	2.4 (1.1–5.1)		2.4 (1.1–5.3)	
Awareness of AG			<0.001		<0.001
Yes (reference)	123				
No	966	2.0 (1.4–3.0)		2.0 (1.3–3.0)	
PainDETECT^c^			<0.001		0.103
<12 (reference)	759				
12–19	254	0.6 (0.5–0.8)		0.8 (0.6–1.1)	
≥19	76	0.3 (0.2–0.6)		0.6 (0.3–1.0)	

^a^ Odds ratio for untreated patients vs. treated patients.

^b^ Calculated using the average 2,021 exchange rate of ¥110 = USD$1.

^c^ Only for the chronic pain survey.

AG, authorized generic drugs; CI, confidence interval; EQ-5D-5l, EuroQol 5 dimensions 5-level; HLS-EU-Q47, European health literacy survey questionnaire; NRS, numerical rating scale; PDAS, pain disability assessment scale.

In treated patients, the main reasons for seeking treatment were “reaching the institution seems easy” (44.0%), followed by “anticipated treatment effect” (39.5%) and “superior expertise” (30.2%) when multiple reasons were allowed, and “reaching the institution seems easy” (28.7%), “anticipated treatment effect” (27.3%), and “superior expertise” (16.3%) when only one reason was allowed (see Supplementary Table S5, Supplemental Digital Content 1).

The association between the awareness rates of generic drugs and AG and healthcare visits in patients with chronic pain is shown in Supplementary Tables S6 and S7, Supplemental Digital Content 1. Although the generic drug awareness rate was similar between the treated and untreated groups, some differences (approximately 10%) were seen between the two groups depending on the item evaluated. The AG awareness rate was 2-fold higher in treated patients (74/484; 15.3%) than in untreated patients (49/605; 8.1%).

#### 3.3.2. Potential migraine

Multivariate logistic regression analysis revealed the factors significantly associated with non-treatment included female (OR: 1.6; 95% CI: 1.2–2.3), less time required to access the nearest medical institution (OR: 0.7; 95% CI: 0.6–0.8), population of municipality of residence <50,000 (OR: 2.2; 95% CI: 1.3–3.8), moderate/severe pain (moderate, OR: 0.6; 95% CI: 0.4–0.9; severe, OR: 0.6; 95% CI: 0.3–0.9), higher ADL (PDAS score ≥10, OR: 0.5; 95% CI: 0.4–0.7), and lower awareness of AG (OR: 2.1; 95% CI: 1.4–3.0) (Table 3).

**Table 3:** Factors associated with non-treatment in patients with potential migraines by univariate and multivariate logistic regression analysis.

	Number of patients	Univariate		Multivariate	
		Odds ratio estimate^a^ (95% CI)	*p* value	Odds ratio estimate^a^ (95% CI)	*p* value
Sex			<0.001		0.004
Male (reference)	372				
Female	560	1.9 (1.4–2.5)		1.6 (1.2–2.3)	
Age (years)			0.253		
20–39 (reference)	444				
40–64	445	1.1 (0.8–1.5)			
≥65	43	0.7 (0.3–1.3)			
Occupation			0.006		0.323
Manual labor (reference)	157				
Customer service, sales	221	1.1 (0.7–1.8)		1.1 (0.7–1.8)	
Office work	294	1.1 (0.7–1.6)		1.1 (0.7–1.8)	
Student	18	3.4 (0.8–15.5)		2.2 (0.4–10.8)	
Unemployed	242	2.0 (1.3–3.3)		1.6 (1.0–2.8)	
Education			0.121		
Junior high school/high school/vocational (reference)	410				
Junior college/university	456	0.7 (0.5–1.0)			
Graduate school	66	0.8 (0.5–1.5)			
Access to a healthcare institution			<0.001		<0.001
Time required to access the nearest medical institution (min) (unit: 10 min.)	932	0.7 (0.6–0.8)		0.7 (0.6–0.8)	
Population of municipality of residence			0.003		0.014
≥100,000 (reference)	551				
50,000–100,000	229	0.9 (0.7–1.3)		1.1 (0.7–1.5)	
<50,000	152	2.2 (1.4–3.6)		2.2 (1.3–3.8)	
Household income [yen (US$)], *n* (%)^b^			0.351		
<1 million [<9,091] (reference)	120				
≥1 to <2 million [≥9,091 to <18,182]	93	0.9 (0.5–1.8)			
≥2 to <3 million [≥18,182 to <27,273]	118	1.0 (0.5–1.8)			
≥3 to <4 million [≥27,273 to <36,364]	113	0.8 (0.4–1.5)			
≥4 to <5 million [≥36,364 to <45,455]	114	1.2 (0.6–2.3)			
≥5 to <6 million [≥45,455 to <54,545]	81	0.8 (0.4–1.5)			
≥6 to <7 million [≥54,545 to <63,636]	55	0.9 (0.4–1.8)			
≥7 to <8 million [≥63,636 to <72,727]	68	0.6 (0.3–1.3)			
≥8 to <10 million [≥72,727 to <90,909]	68	0.9 (0.5–1.9)			
≥10 million [≥90,909]	102	0.5 (0.3–1.0)			
Status of migraine (NRS)			<0.001		0.009
Mild (reference)	486				
Moderate	337	0.6 (0.4–0.8)		0.6 (0.4–0.9)	
Severe	109	0.5 (0.3–0.7)		0.6 (0.3–0.9)	
Duration (months)	932	1.0 (1.0–1.0)	0.350		
Health literacy (HLS-EU-Q47)			0.305		
0–25 (insufficient) (reference)	346				
25–33 (problematic)	254	0.9 (0.6–1.4)			
33–42 (sufficient)	124	0.7 (0.4–1.0)			
42–50 (excellent)	59	0.8 (0.4–1.4)			
Quality of life (EQ-5D-5l)					
Utility index (unit: 0.1)	932	1.2 (1.1–1.3)	<0.001		
VAS (unit: 10)	932	1.1 (1.0–1.2)	0.010		
Activities of daily living (PDAS)			<0.001		<0.001
<10 (reference)	548				
≥10	384	0.4 (0.3–0.6)		0.5 (0.4–0.7)	
Awareness of generic drugs			0.017		0.058
Yes (reference)	807				
No	125	1.8 (1.1–3.0)		1.7 (1.0–3.0)	
Awareness of AG			<0.001		<0.001
Yes (reference)	184				
No	748	3.4 (2.4–4.7)		2.1 (1.4–3.0)	

^a^ Odds ratio for untreated patients vs. treated patients.

^b^ Calculated using the average 2021 exchange rate of ¥110 = USD$1.

AG, authorized generic drugs; CI, confidence interval; EQ-5D-5l, EuroQol 5 dimensions 5-level; HLS-EU-Q47, European health literacy survey questionnaire; NRS, numerical rating scale; PDAS, pain disability assessment scale.

The main reasons for seeking treatment in the treated group were “anticipated treatment effect” (37.6%), “reaching the institution seems easy” (37.1%), and “healthcare professionals were kind and nice during my consultation” (30.0%) when multiple reasons were allowed, and “anticipated treatment effect” (23.6%), “reaching the institution seems easy” (19.8%), and “superior expertise” (16.0%) when only one reason was allowed (see Supplementary Table S8, Supplemental Digital Content 1).

The awareness of generic drugs or AG based on the detailed questions is shown in Supplementary Tables S6 and S7, Supplemental Digital Content 1. Generic drug awareness rates were similar between the treated and untreated groups, although some differences (approximately 5%) were observed depending on the question evaluated. Both generic drug and AG awareness rates tended to be higher in the treated group, with AG awareness rates being 2-fold higher in treated vs. untreated patients (untreated vs. treated:591/695, 85.0% vs. 216/237, 91.1% and 99/695, 14.2% vs. 85/237, 35.9%, respectively).

## 4. Discussion

There is little information on why more than half of the patients with chronic pain or migraines do not seek treatment (9, 13). The present study clarifies this issue with the expectation that information may contribute to improved access to healthcare.

Among patients with chronic pain or migraines, two of the three most common reasons given for non-treatment were “my pain is tolerable” and “expense.” Among patients with chronic pain, the third most common reason given for non-treatment was “I do not believe that my pain can be resolved through outpatient visits.” For migraine patients, the main reason for non-treatment was “I can manage my pain with over-the-counter drugs.” These findings suggest that some patients with chronic pain perceive pain as a symptom that cannot be treated despite seeking medical care, and those with migraines tend to manage their pain by self-medication and therefore do not seek treatment at medical institutions. Medical examination and treatment under medical consultation are required for both chronic pain and migraines because pain can sometimes be a signal of an underlying and undiagnosed severe disease and, if left untreated, may lead to prolonged pain and disability (28). Therefore, patients with chronic pain and migraines require more information regarding pain and related diseases as well as the risks of not seeking medical care. In addition, the issue of medication overuse for headaches should be considered, which can occur after the continuous use of medications, including over-the-counter drugs, and may worsen pain (29, 30).

Patients' low expectations of addressing their pain through outpatient treatment were the third and fourth most common reasons for not seeking treatment for chronic pain and migraines, respectively. This low expectation may be a result of patients' distrust of treatment based on previous experiences with a lack of satisfaction with treatment. A previous survey showed that treatment satisfaction was low in patients with chronic pain (31). Additionally, the lack of appropriate treatment for neuropathic or psychogenic pain is associated with a chronic pain course (31). Considering these findings, along with the present study's findings, many patients who receive treatment for chronic pain may become dissatisfied with treatment, choose to discontinue treatment, and stop seeking medical attention in general, particularly in appropriate treatment facilities. Therefore, these patients need to not only be educated about their condition and risks of non-treatment but also receive counselling about realistic treatment expectations, be informed about different treatment options, and be provided guidance on where to access appropriate treatment facilities.

In general, financial factors are one of the barriers to healthcare access (15), and in the present study, they were among the most frequent reasons for not seeking treatment for chronic pain and migraine. In Japan, the proportion of an individual's out-of-pocket burden within Japan's national health insurance system is 10%–30%, depending on the age bracket, and no medical expenses are paid by individuals covered by the livelihood protection system (32). Nonetheless, the economic burden appeared to affect patients' care-seeking behaviors in this study. Increasing the awareness of inexpensive treatment options may contribute to improved care-seeking behaviors.

Multivariate regression analysis showed that factors such as younger age, less time required to access the nearest medical institution, lower pain severity, higher ADL scores, and lower awareness of generic drugs and AG were associated with nontreatment. Our data are consistent with those of previously published reports on age and pain severity (33, 34); patients may not seek treatment unless their pain and health conditions worsen.

An important finding of the present study is that the awareness of both generic drugs and AG is associated with healthcare access. An authorized generic (AG) is a pharmaceutical product that is identical to a brand name drug but marketed as a generic version without the brand label. Untreated patients with chronic pain tended to be less aware of generic drugs than treated patients. It is possible that the treated patients were more familiar with generic drugs than the untreated patients because they obtained information during their healthcare visits. Regarding awareness of AG, there was a 2-fold difference in overall recognition between treated and untreated patients with chronic pain or migraine. The awareness of AG was much lower than that of generic drugs. Based on this, we consider that the concept of AG as a drug product that is unbranded but is otherwise identical to the brand-name drug product (35) is not widely recognized in the broader society in Japan because it is relatively new. Considering that concerns about treatment costs were expressed, greater awareness of generic drugs and AG, which are inexpensive treatment options, could have a positive effect on medical care-seeking behavior. Further research is needed to determine whether awareness of low-cost treatment options promotes positive behavioral changes to seek medical consultation among untreated patients.

Interestingly, in the present study, the time required to access the nearest medical institution was shorter in untreated patients. The reason for this is unclear, but information about appropriate treatment facilities for pain is important for improving treatment access. In the migraine group, factors associated with non-treatment included female sex and living in a municipality with a population of <50,000 people. Although it is assumed that women are generally more likely than men to consult general practitioners for all symptoms and conditions, the difference is less clear for headache and back pain (36). Municipalities with smaller populations have fewer specialized medical institutions; therefore, patients with uncommon conditions, such as migraines, may not know where to seek treatment, which may have contributed to the lower consultation rate in these regions.

Health problems among workers have a negative impact on society. Previous studies have investigated the economic burden of presenteeism due to various health problems (37–39), and musculoskeletal pain, mental illnesses, and headaches were found to be the health conditions with the highest cost loss of presenteeism (37). In the present study, absenteeism and presenteeism were higher in treated vs. untreated patients for both chronic pain and migraine, and cost loss was greater in treated vs. untreated patients, regardless of absenteeism or presenteeism. The reason for this may be that the treated patients had higher pain intensity than the untreated patients. A previous study reported that pain severity is associated with increased absenteeism, presenteeism, and healthcare use (40). The QOL and ADL scores tended to be lower in treated patients in this study. Therefore, it is conceivable that appropriate treatment before the disease worsens can reduce the impact on socioeconomic loss. However, further research is needed to evaluate this. In addition, this study found that the cost lost based on presenteeism, which is difficult for employers to notice, was greater than the cost lost based on absenteeism for both chronic pain and migraines, regardless of the treatment status (untreated or treated). This is consistent with a previous report showing that pain affects presenteeism more than absenteeism (41).

### 4.1. Limitations

This study has some limitations, including those inherent to the survey design, such as the possibility of recall bias. In the population with potential migraine, the diagnosis was not made by a physician but was based on simple migraine questionnaire results. Therefore, patients with potential migraine in this study did not have a confirmed migraine diagnosis; thus, some patients may not have met the criteria for migraines and instead may have suffered from headaches or diseases other than migraines. As this study used an internet questionnaire survey, it was difficult to determine whether the participants answered truthfully. Therefore, the data entered in the questionnaire may not have been reliable. Finally, the questionnaire survey targeted a panel maintained by the survey agency, which may have led to bias; therefore, the population analyzed may not be representative of the general population.

## 5. Conclusion

The current study revealed the reasons for and factors associated with barriers to healthcare access in patients with chronic pain or migraines. Our findings suggest that to improve healthcare access in patients with chronic pain or migraines, it is necessary to educate patients about the risks associated with their pain and underlying disease, the availability of inexpensive treatment options, and the location of appropriate treatment facilities.

## Data Availability

The original contributions presented in the study are included in the article/**Supplementary Material**, further inquiries can be directed to the corresponding author.
