# Supplementary material for: Barriers to healthcare access in patients with chronic pain or potential migraine in Japan: a cross-sectional internet survey
Source: Front Pain Res (Lausanne). 2023 Oct 3;4:1271438. doi: 10.3389/fpain.2023.1271438 (PMC10579894; doi:10.3389/fpain.2023.1271438)
Supplement: Supplementary file 1 [file Presentation1.pptx]

## Slide 1
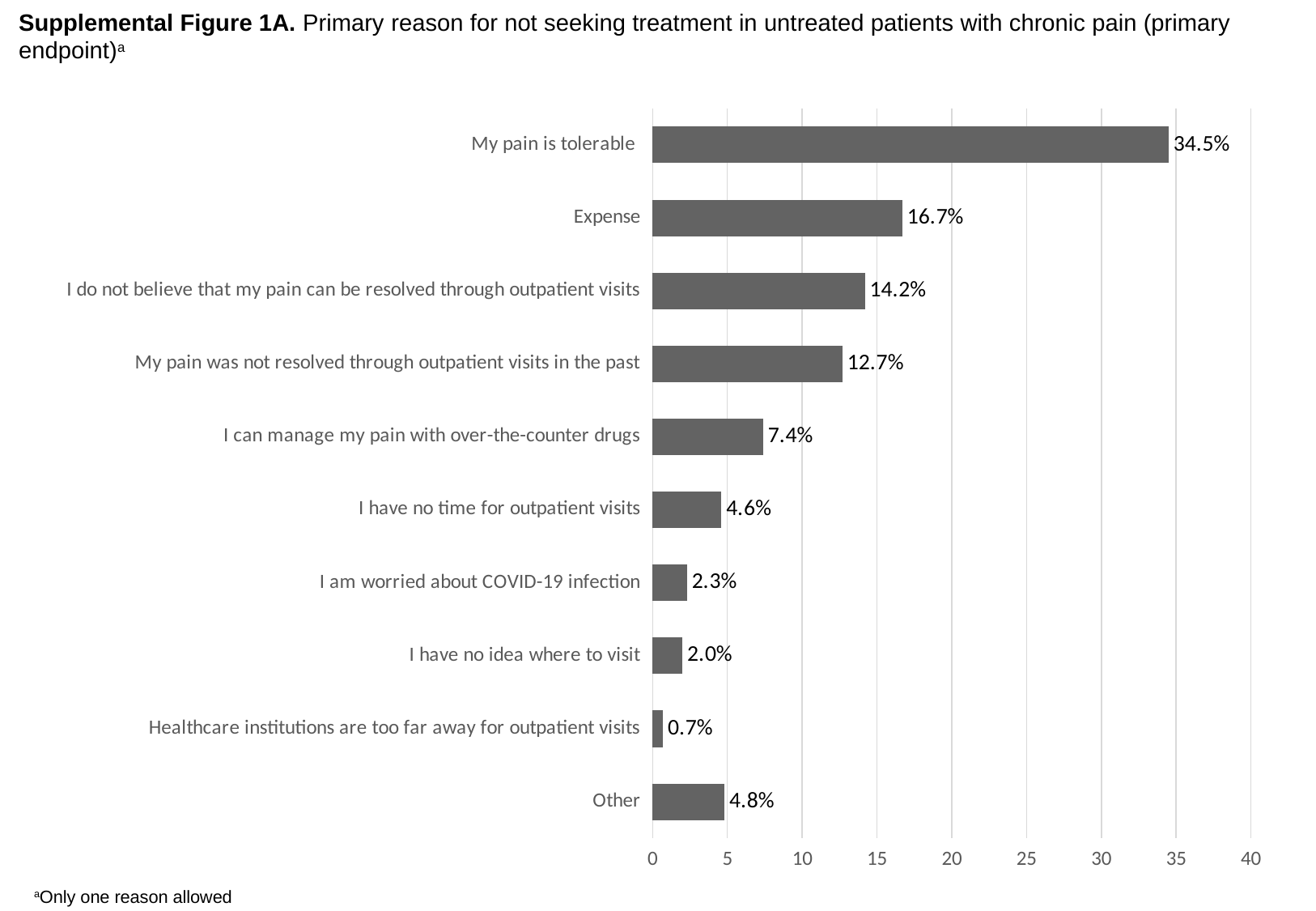

Supplemental Figure 1A. Primary reason for not seeking treatment in untreated patients with chronic pain (primary endpoint)a
### Chart
| Category | Chronic pain |
|---|---|
| Other | 4.8 |
| Healthcare institutions are too far away for outpatient visits | 0.7 |
| I have no idea where to visit | 2.0 |
| I am worried about COVID-19 infection | 2.3 |
| I have no time for outpatient visits | 4.6 |
| I can manage my pain with over-the-counter drugs | 7.4 |
| My pain was not resolved through outpatient visits in the past | 12.7 |
| I do not believe that my pain can be resolved through outpatient visits | 14.2 |
| Expense | 16.7 |
| My pain is tolerable | 34.5 |aOnly one reason allowed

## Slide 2
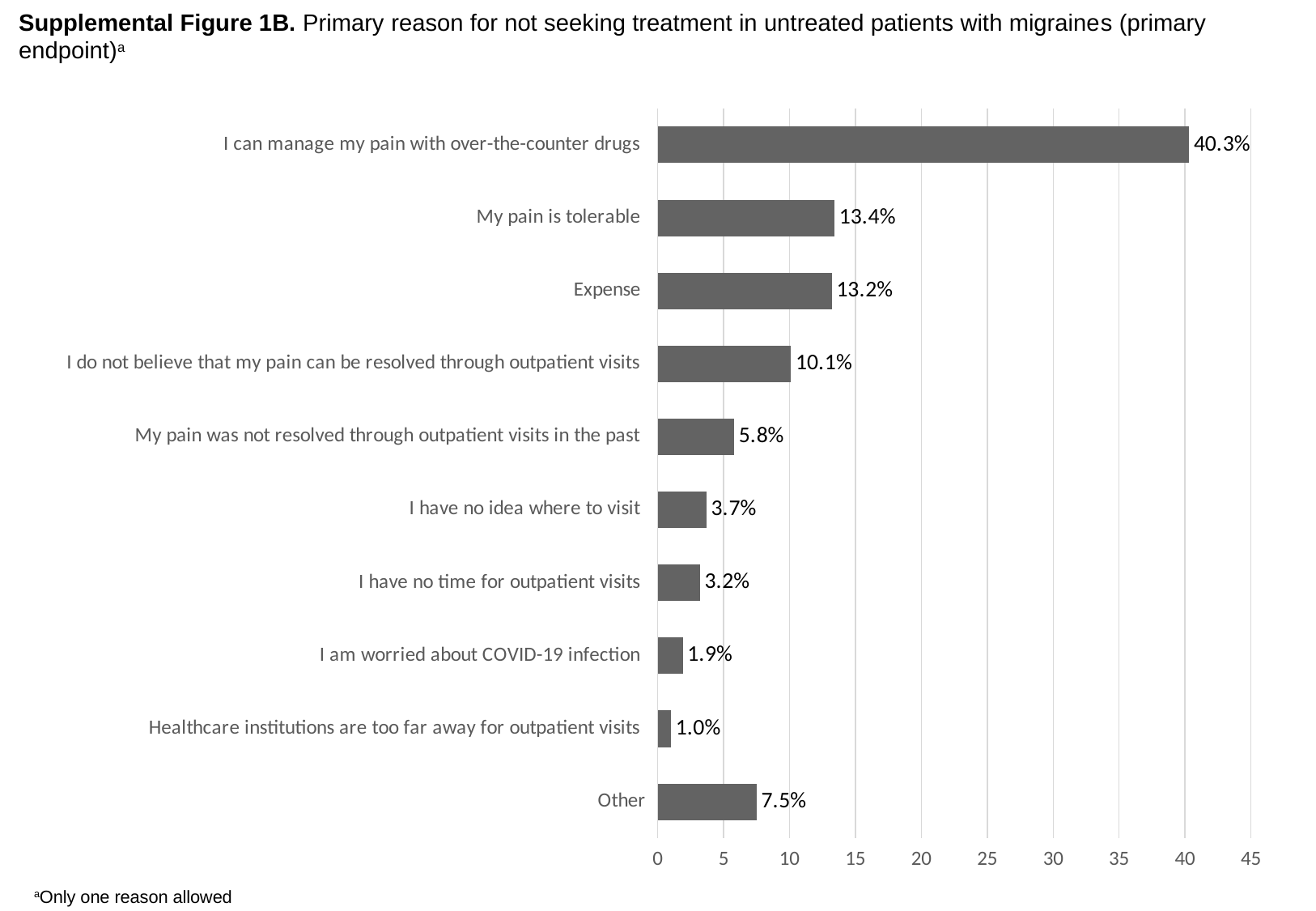

Supplemental Figure 1B. Primary reason for not seeking treatment in untreated patients with migraines (primary endpoint)a
### Chart
| Category | % |
|---|---|
| Other | 7.5 |
| Healthcare institutions are too far away for outpatient visits | 1.0 |
| I am worried about COVID-19 infection | 1.9 |
| I have no time for outpatient visits | 3.2 |
| I have no idea where to visit | 3.7 |
| My pain was not resolved through outpatient visits in the past | 5.8 |
| I do not believe that my pain can be resolved through outpatient visits | 10.1 |
| Expense | 13.2 |
| My pain is tolerable | 13.4 |
| I can manage my pain with over-the-counter drugs | 40.3 |aOnly one reason allowed
