# Supplementary material for: Barriers to healthcare access in patients with chronic pain or potential migraine in Japan: a cross-sectional internet survey
Source: Front Pain Res (Lausanne). 2023 Oct 3;4:1271438. doi: 10.3389/fpain.2023.1271438 (PMC10579894; doi:10.3389/fpain.2023.1271438)
Supplement: Supplementary file 2 [file Datasheet1.docx]

**Supplemental Digital Content 1**

**Supplementary Methods**

Evaluation methods for questionnaires used in this survey

***Simple migraine questionnaire***^10^

Simple migraine questionnaire is a simple migraine screening instrument. There are four options for answering the questionnaire for all items: ‘never’, ‘rarely’, ‘sometimes’, and ‘more than half the time’. Participants who answered “sometimes” or “half the time or more” for two or more items in the simple migraine questionnaire are regarded as migraine patients. This is shown in **Supplemental Table 2.**

***Numeric rating scale (NRS)^4^***

Numeric rating scale (NRS) for pain is a measure of pain intensity. The pain NRS is a single 11-point numeric scale, with 0 representing no pain and 10 representing pain as bad as you can imagine. NRS values of 0–4, 5–7, and 8–10 are classified as mild, moderate, and severe, respectively.

***European Health Literacy Survey Questionnaire^5^***

European Health Literacy Survey Questionnaire is a questionnaire of ability in understanding health-related issues, and difficult situations that might easily arise without adequate health literacy. This questionnaire contained 47 health literacy related items. Health literacy levels of 0–25, 25–33, 33–42, and 42–50 are classified as inadequate, problematic, sufficient, and excellent, respectively.

***EuroQol 5 dimensions 5-level (EQ-5D-5L)^2^***

EuroQol 5 dimensions 5-level (EQ-5D-5L) consists of an EQ-5D descriptive section and the EQ visual analogue scale (EQ VAS). The descriptive system covers five aspects of quality of life: mobility, self-care, usual activities, pain/discomfort, and anxiety/depression. A single-digit number is derived for each dimension, and the patient’s health status is described using the resulting five-digit number. The EQ VAS is a measure of a quantitative index of health status, reflecting patients’ own assessments.

***Pain Disability Assessment Scale (PDAS)^1^***

Pain Disability Assessment Scale (PDAS) is a self-reported scale that assesses the degree to which chronic pain interferes with various daily activities, such as physical exercise and mobility. PDAS scores <10 and ≥10 are classified as healthy and with chronic pain, respectively.

***Work productivity and activity impairment (WPAI)^6^***

Work productivity and activity impairment (WPAI) is a simple questionnaire to measure the impact of health problems on absenteeism, presenteeism, overall work performance, and non-work activities. WPAI yields four types of scores: absenteeism, presenteeism, work productivity loss, and activity impairment. WPAI outcomes are expressed as impairment percentages, with higher numbers indicating greater impairment and less productivity.

***PainDETECT^3^***

PainDETECT is a simple screening tool to detect neuropathic pain (NeP) in chronic LBP patients. PainDETECT score of 0–12 points is classified as nociceptive pain with almost no possibility of NeP; 13–18 points is classified as having some factors of neuropathic pain; 19–38 points is classified as neuropathic pain.

***Migraine Disability Assessment Scale^7–9^***

Migraine Disability Assessment is a brief, self-administered questionnaire that quantifies headache-related disability over a 3 month period; it includes five questions regarding days of activity limitations in work, chores, and non-work activities, to score the level of disability from headaches. Scores of 0–5, 6–10, 11–20, and 21 are classified as little or no disability, mild disability, moderate disability, and severe disability, respectively.

**References**

1. Arimura T, Komiyama H, Hosoi M. Pain Disability Assessment Scale (PDAS)-A Simplified Scale for Clinical Use. Jap J Behav Ther 1997;23:1.
2. EQ-5D-5L | About. Available at: <https://euroqol.org/eq-5d-instruments/eq-5d-5l-about/>
3. Freynhagen R, Baron R, Gockel U, Tölle TR. PainDETECT. Curr Med Res Opin 2006;22:1911–20.
4. McCaffery M, Beebe A. Pain: Clinical manual for nursing practice, Mosby St. Louis, MO; 1989.
5. Nakayama K, Osaka W, Togari T, Ishikawa H, Yonekura Y, Sekido A, Matsumoto M. Comprehensive health literacy in Japan is lower than in Europe: a validated Japanese-language assessment of health literacy. BMC Public Health 2015;15:505.
6. Reilly MC, Zbrozek AS, Dukes EM. The validity and reproducibility of a work productivity and activity impairment instrument. Pharmacoeconomics 1993;4:353–365.
7. Stewart WF, Lipton RB, Kolodner K, Liberman J, Sawyer J. Reliability of the migraine disability assessment score in a population-based sample of headache sufferers. Cephalalgia. 1999;19:107–14; discussion 74.
8. Stewart WF, Lipton RB, Kolodner KB, Sawyer J, Lee C, Liberman JN. Validity of the Migraine Disability Assessment (MIDAS) score in comparison to a diary-based measure in a population sample of migraine sufferers. Pain 2000;88(1):41–52.
9. Stewart WF, Lipton RB, Whyte J, Dowson A, Kolodner K, Liberman JN, Sawyer J. An international study to assess reliability of the Migraine Disability Assessment (MIDAS) score. Neurology 1999;53:988–94.
10. Takeshima T, Sakai F, Suzuki N, Shimizu T, Igarashi H, Araki N, Manaka S, Nakashima K, Hashimoto Y, Iwata M, Fukuuchi Y. A simple migraine screening instrument: validation study in Japan. Japanese Journal of Headache 2015;42:134–43.

**Supplemental Table 1.** Simple chronic pain screening questionnaire

| **Question Text and Response Options** | | |
| --- | --- | --- |
| Q1. Please tell us your age. | | |
|  | 1 | ________ years old |
| Q2. Have you had any body pain (other than headache) for more than 3 months? | | |
|  | 1 | Yes |
|  | 2 | No |
| Q3. Please tell us about the body pain (excluding headache) that you answered about in Q2. Is the pain caused by cancer? | | |
|  | 1 | Yes |
|  | 2 | No |
| Q4. Please tell us about the body pain (excluding headache) that you answered about in Q2. Have you been feeling that pain in the last month? | | |
|  | 1 | Yes |
|  | 2 | No |
| Q5. Please tell us about the body pain (excluding headache) that you answered about in Q2. On a scale of 11 grades from 0 to 10, how is your current pain level? Please answer 0 as no pain at all and 10 as the worst possible pain. | | |
|  |  | 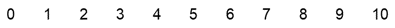 |
| Q6. Have you visited a healthcare institution* within the past year due to your current pain? *Visiting a medical facility: In addition to hospitals and clinics, this includes orthopedic clinics, osteopathic clinics, acupuncture and moxibustion clinics, chiropractic clinics, and massage clinics. However, pharmacies and drug stores are not included. | | |
|  | 1 | Yes |
|  | 2 | No |

**Supplemental Table 2.** Simple migraine screening questionnaire

| **Question Text and Response Options** | | |
| --- | --- | --- |
| Q1. Please tell us your age. | | |
|  | 1 | ________ years old |
| Q2. How often was your headache worsened by everyday activities such as walking or climbing/descending stairs, or how often was it easier to stay still than move? | | |
|  | 1 | Never |
|  | 2 | Rarely |
|  | 3 | Sometimes |
|  | 4 | More than half the time |
| Q3. How often did you experience nausea or an upset stomach with your headache? | | |
|  | 1 | Never |
|  | 2 | Rarely |
|  | 3 | Sometimes |
|  | 4 | More than half the time |
| Q4. With headache, how often have you experienced glare from light that would normally go unnoticed? | | |
|  | 1 | Never |
|  | 2 | Rarely |
|  | 3 | Sometimes |
|  | 4 | More than half the time |
| Q5. With headache, how often have you found smells unpleasant? | | |
|  | 1 | Never |
|  | 2 | Rarely |
|  | 3 | Sometimes |
|  | 4 | More than half the time |
| Q6. Have you visited a healthcare institution* within the past year due to your current headache?  *Visiting a medical facility: In addition to hospitals and clinics, this includes orthopedic clinics, osteopathic clinics, acupuncture and moxibustion clinics, chiropractic clinics, and massage clinics. However, pharmacies and drug stores are not included. | | |
|  | 1 | Yes |
|  | 2 | No |

**Supplemental Table 3.** Main chronic pain questionnaire

| **Question Text and Response Options** | | |
| --- | --- | --- |
| Q1. Please tell us about your job. | | |
|  | 1 | Manual labor (work that involves handling heavy objects and moving the body) |
|  | 2 | Customer service, sales (light work or work involving movement) |
|  | 3 | Office work (office-based, such as clerical work) |
|  | 4 | Student |
|  | 5 | Unemployed |
| Q2. Please tell us about your final educational background. | | |
|  | 1 | Junior high school/high school/vocational |
|  | 2 | Junior college/university |
|  | 3 | Graduate school |
| Q3. What is the population size of your area? | | |
|  | 1 | ≥100,000 |
|  | 2 | 50,000–100,000 |
|  | 3 | <50,000 |
| Q4. How long does it take to get to the nearest healthcare institution*?  *Healthcare institution: Hospitals, clinics, osteopathic clinics, osteopathic clinics, acupuncture and moxibustion clinics, chiropractic clinics, massages, and other places that are likely to be institutions for current pain treatment (excluding pharmacies and drug stores) | | |
|  | 1 | _________ minutes |
| Q5. Please select your current household income (¥) from the options below. | | |
|  | 1 | No income |
|  | 2 | <1 million |
|  | 3 | ≥1 to <2 million |
|  | 4 | ≥2 to <3 million |
|  | 5 | ≥3 to <4 million |
|  | 6 | ≥4 to <5 million |
|  | 7 | ≥5 to <6 million |
|  | 8 | ≥6 to <7 million |
|  | 9 | ≥7 to <8 million |
|  | 10 | ≥8 to <10 million |
|  | 11 | ≥10 million |
| Q6. Please tell us about the pain that you previously answered about.  How long has the pain been going on? | | |
|  | 1 | _______ years _________ months |
| Q7. (This question is only for those who have visited a healthcare institution.)  Please continue to tell us about your body pain (excluding headache).  What made you start visiting the healthcare institution* for pain treatment?  Please select all that apply.  *Visiting a medical facility: In addition to hospitals and clinics, this includes orthopedic clinics, osteopathic clinics, acupuncture and moxibustion clinics, chiropractic clinics, and massage clinics. However, pharmacies and drug stores are not included. | | |
|  | 1 | The waiting time seemed short. |
|  | 2 | Access to the institution seemed easy. |
|  | 3 | The treatment cost seemed low. |
|  | 4 | They have excellent expertise. |
|  | 5 | Healthcare professional’s kindness. |
|  | 6 | Therapeutic effects were expected. |
|  | 7 | Other |
| Q8. (This question is only for those who have visited a healthcare institution.)  Please select the number one reason that made you visit the institution for pain treatment.  (Only the options selected in Q7 are shown) | | |
|  | 1 | The waiting time seemed short. |
|  | 2 | Access to the institution seemed easy. |
|  | 3 | The treatment cost seemed low. |
|  | 4 | They have excellent expertise. |
|  | 5 | Healthcare professional’s kindness. |
|  | 6 | Therapeutic effects were expected. |
|  | 7 | Other |
| Q9. (This question is only for those who have not visited a healthcare institution.)  Please continue to tell us about body pain (excluding headache).  Please tell us the reason why you have not visited a healthcare institution* in spite of your pain.  Please select all that apply.  *Visiting a medical facility: In addition to hospitals and clinics, this includes orthopedic clinics, osteopathic clinics, acupuncture and moxibustion clinics, chiropractic clinics, and massage clinics. However, pharmacies and drug stores are not included. | | |
|  | 1 | My pain is tolerable. |
|  | 2 | I visited a healthcare institution in the past, but my pain did not resolve. |
|  | 3 | Due to the cost. |
|  | 4 | I don't have time to visit a healthcare institution. |
|  | 5 | I can use over-the-counter medicines. |
|  | 6 | I worry about COVID-19 infection. |
|  | 7 | I don't think I can get better visiting a healthcare institution. |
|  | 8 | I don't know what kind of doctor I should see. |
|  | 9 | It is too far to reach a healthcare institution. |
|  | 10 | Other |
| Q10. (This question is only for those who have not visited a healthcare institution.)  Please select the number one reason why you do not go to the hospital* in spite of your pain.  *Visiting a medical facility: In addition to hospitals and clinics, this includes orthopedic clinics, osteopathic clinics, acupuncture and moxibustion clinics, chiropractic clinics, and massage clinics. However, pharmacies and drug stores are not included.  (Only the options selected in Q7 are shown) | | |
|  | 1 | My pain is tolerable. |
|  | 2 | I visited a healthcare institution in the past, but my pain did not resolve. |
|  | 3 | Due to the cost. |
|  | 4 | I don't have time to visit a healthcare institution. |
|  | 5 | I can use over-the-counter medicines. |
|  | 6 | I worry about COVID-19 infection. |
|  | 7 | I don't think I can get better visiting a healthcare institution. |
|  | 8 | I don't know what kind of doctor I should see. |
|  | 9 | It is too far to reach a healthcare institution. |
|  | 10 | Other |
| Q11. (This question is only for those who have not visited a healthcare institution.)  Which of the following factors would make you visit a healthcare institution for pain treatment?  Please select all that apply.  *Visiting a medical facility: In addition to hospitals and clinics, this includes orthopedic clinics, osteopathic clinics, acupuncture and moxibustion clinics, chiropractic clinics, and massage clinics. However, pharmacies and drug stores are not included. | | |
|  | 1 | It does not take much time (e.g., short waiting time). |
|  | 2 | Physical distance is good (e.g., healthcare institution is located nearby). |
|  | 3 | High-quality treatment at minimum cost is available. |
|  | 4 | Healthcare professional’s kindness during consultation. |
|  | 5 | Other |
| Q12. (This question is only for those who have not visited a healthcare institution.)  Which of the following factors would make you visit a healthcare institution for pain treatment?  Please select the number one factor.  *Visiting a medical facility: In addition to hospitals and clinics, this includes orthopedic clinics, osteopathic clinics, acupuncture and moxibustion clinics, chiropractic clinics, and massage clinics. However, pharmacies and drug stores are not included.  (Only the options selected in Q11 are shown) | | |
|  | 1 | It does not take much time (e.g., short waiting time). |
|  | 2 | Physical distance is good (e.g., healthcare institution is located nearby). |
|  | 3 | High-quality treatment at minimum cost is available. |
|  | 4 | Healthcare professional’s kindness during consultation. |
|  | 5 | Other |
| Q13. (This question is only for those who have not visited a healthcare institution.)  Are you currently using over-the-counter medications to treat chronic pain? | | |
|  | 1 | I'm using them. |
|  | 2 | I'm not using them. |
| Q14. Do you know about generic drugs? | | |
|  | 1 | I know about them. |
|  | 2 | I don’t know about them. |
| Q15. (This question is only for those who answered "I know about them" in Q14.)  Please select all you know about generic drugs. | | |
|  | 1 | The drug cost is lower compared with branded drugs. |
|  | 2 | Efficacy is equivalent to branded drugs. |
|  | 3 | Drugs manufactured and sold by another manufacturer after the branded drugs’ patent has expired. |
|  | 4 | Drugs approved by the country (Ministry of Health, Labour and Welfare). |
|  | 5 | Medical expenses (pharmaceutical expenses) can be saved, helping to maintain the universal health insurance system. |
|  | 6 | Some drugs do not have generic drugs. |
|  | 7 | Equivalent quality, efficacy and safety to branded drugs. |
|  | 8 | Excipients may differ from branded drugs. |
|  | 9 | Depending on the drugs, there are multiple generic drugs for one brand name drug. |
|  | 10 | Because of advances in formulation technology and ingenuity in formulation development by manufacturers, some have been improved in terms of color, shape, taste, etc. to make them easier to take. |
|  | 11 | In some cases, the manufacture and sale of branded drugs has ended, and only generic drugs are available. |
| Q16. Do you know authorized generic (AG) drugs? | | |
|  | 1 | I know about them. |
|  | 2 | I don’t know about them. |
| Q17. (This question is only for those who answered "I know about them" in Q16.)  Do you know that AG drugs are generic drugs manufactured under license from the branded drug manufacturer? | | |
|  | 1 | I know this. |
|  | 2 | I don’t know this. |
| Q18. (This question is only for those who answered "I know about them" in Q16.)  Do you know that AG drugs are basically the same as branded drugs except for the name, appearance and packaging?  (The active ingredient, drug substance, excipients, manufacturing method, manufacturing technology, etc. are all the same as the branded drug. *The manufacturing plant may differ.) | | |
|  | 1 | I know this. |
|  | 2 | I don’t know this. |
| Q19. This question is only for those who answered "I know about them" in Q16.)  Regarding AG drugs, do you know that generic drugs, including AG drugs, are less expensive than branded drugs due to less development costs and time? | | |
|  | 1 | I know this. |
|  | 2 | I don’t know this. |
| Q20. HLS-EU-Q47 | | |
| Q21. EQ-5D-5L | | |
| Q22. PDAS | | |
| Q23. WPAI | | |
| Q24. PainDETECT | | |

**Supplemental Table 4.** Main migraine questionnaire

| **Question Text and Response Options** | | | |
| --- | --- | --- | --- |
| Q1. Please tell us about your job. | | | |
|  | 1 | Manual labor (work that involves handling heavy objects and moving the body) | |
|  | 2 | Customer service, sales (light work or work involving movement) | |
|  | 3 | Office work (office-based, such as clerical work) | |
|  | 4 | Student | |
|  | 5 | Unemployed | |
| Q2. Please tell us about your final educational background. | | | |
|  | 1 | Junior high school/high school/vocational | |
|  | 2 | Junior college/university | |
|  | 3 | Graduate school | |
| Q3. What is the population size of your area? | | | |
|  | 1 | ≥100,000 | |
|  | 2 | 50,000–100,000 | |
|  | 3 | <50,000 | |
| Q4. How long does it take to get to the nearest healthcare institution*?  *Healthcare institution: Hospitals, clinics, osteopathic clinics, osteopathic clinics, acupuncture and moxibustion clinics, chiropractic clinics, massages, and other places that are likely to be institutions for current headache treatment (excluding pharmacies and drug stores) | | | |
|  | 1 | _________ minutes | |
| Q5. Please select your current household income (¥) from the options below. | | | |
|  | 1 | No income | |
|  | 2 | <1 million | |
|  | 3 | ≥1 to <2 million | |
|  | 4 | ≥2 to <3 million | |
|  | 5 | ≥3 to <4 million | |
|  | 6 | ≥4 to <5 million | |
|  | 7 | ≥5 to <6 million | |
|  | 8 | ≥6 to <7 million | |
|  | 9 | ≥7 to <8 million | |
|  | 10 | ≥8 to <10 million | |
|  | 11 | ≥10 million | |
| Q6. Please tell us about the headache that you previously answered about.  How long has the headache been going on? | | | |
|  | 1 | _______ years _________ months | |
| Q7. Please tell us about the headache that you answered about. On a scale of 11 grades from 0 to 10, how is your current headache level? Please answer 0 as no headache at all and 10 as the worst possible headache. | | | |
|  |  | | 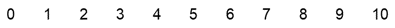 |
| Q8. (This question is only for those who have visited a healthcare institution.)  Please continue to tell us about your headache.  What made you start visiting the healthcare institution* for headache treatment?  Please select all that apply.  *Visiting a medical facility: In addition to hospitals and clinics, this includes orthopedic clinics, osteopathic clinics, acupuncture and moxibustion clinics, chiropractic clinics, and massage clinics. However, pharmacies and drug stores are not included. | | | |
|  | 1 | The waiting time seemed short. | |
|  | 2 | Access to the institution seemed easy. | |
|  | 3 | The treatment cost seemed low. | |
|  | 4 | They have excellent expertise. | |
|  | 5 | Healthcare professional’s kindness. | |
|  | 6 | Therapeutic effects were expected. | |
|  | 7 | Other | |
| Q9. (This question is only for those who have visited a healthcare institution.)  Please select the number one reason that made you visit the institution for headache treatment.  (Only the options selected in Q8 are shown) | | | |
|  | 1 | The waiting time seemed short. | |
|  | 2 | Access to the institution seemed easy. | |
|  | 3 | The treatment cost seemed low. | |
|  | 4 | They have excellent expertise. | |
|  | 5 | Healthcare professional’s kindness. | |
|  | 6 | Therapeutic effects were expected. | |
|  | 7 | Other | |
| Q10. (This question is only for those who have not visited a healthcare institution.)  Please continue to tell us about your headache.  Please tell us the reason why you have not visited a healthcare institution* in spite of your headache.  Please select all that apply.  *Visiting a medical facility: In addition to hospitals and clinics, this includes orthopedic clinics, osteopathic clinics, acupuncture and moxibustion clinics, chiropractic clinics, and massage clinics. However, pharmacies and drug stores are not included. | | | |
|  | 1 | I can use over-the-counter medicines. | |
|  | 2 | My headache is tolerable. | |
|  | 3 | Due to the cost. | |
|  | 4 | I visited a healthcare institution in the past, but my pain didn’t resolve. | |
|  | 5 | I worry about COVID-19 infection. | |
|  | 6 | I don't have time to visit a healthcare institution. | |
|  | 7 | It is too far to reach a healthcare institution. | |
|  | 8 | I don't know what kind of doctor I should see. | |
|  | 9 | I don't think I can get better visiting a healthcare institution. | |
|  | 10 | Other | |
| Q11. (This question is only for those who have not visited a healthcare institution.)  Please select the number one reason why you do not go to the hospital* in spite of your headache.  *Visiting a medical facility: In addition to hospitals and clinics, this includes orthopedic clinics, osteopathic clinics, acupuncture and moxibustion clinics, chiropractic clinics, and massage clinics. However, pharmacies and drug stores are not included.  (Only the options selected in Q10 are shown) | | | |
|  | 1 | I can use over-the-counter medicines. | |
|  | 2 | My headache is tolerable. | |
|  | 3 | Due to the cost. | |
|  | 4 | I visited a healthcare institution in the past, but my pain didn’t resolve. | |
|  | 5 | I worry about COVID-19 infection. | |
|  | 6 | I don't have time to visit a healthcare institution. | |
|  | 7 | It is too far to reach a healthcare institution. | |
|  | 8 | I don't know what kind of doctor I should see. | |
|  | 9 | I don't think I can get better visiting a healthcare institution. | |
|  | 10 | Other | |
| Q12. (This question is only for those who have not visited a healthcare institution.)  Which of the following factors would make you visit a healthcare institution for headache treatment?  Please select all that apply.  *Visiting a medical facility: In addition to hospitals and clinics, this includes orthopedic clinics, osteopathic clinics, acupuncture and moxibustion clinics, chiropractic clinics, and massage clinics. However, pharmacies and drug stores are not included. | | | |
|  | 1 | It does not take much time (e.g., short waiting time). | |
|  | 2 | Physical distance is good (e.g., healthcare institution is located nearby). | |
|  | 3 | High-quality treatment at minimum cost is available. | |
|  | 4 | Healthcare professional’s kindness during consultation. | |
|  | 5 | Other | |
| Q13. (This question is only for those who have not visited a healthcare institution.)  Which of the following factors would make you visit a healthcare institution for headache treatment?  Please select the number one factor.  *Visiting a medical facility: In addition to hospitals and clinics, this includes orthopedic clinics, osteopathic clinics, acupuncture and moxibustion clinics, chiropractic clinics, and massage clinics. However, pharmacies and drug stores are not included.  (Only the options selected in Q11 are shown) | | | |
|  | 1 | It does not take much time (e.g., short waiting time). | |
|  | 2 | Physical distance is good (e.g., healthcare institution is located nearby). | |
|  | 3 | High-quality treatment at minimum cost is available. | |
|  | 4 | Healthcare professional’s kindness during consultation. | |
|  | 5 | Other | |
| Q14. (This question is only for those who have not visited a healthcare institution.)  Are you currently using over-the-counter medications to treat headache? | | | |
|  | 1 | I'm using them. | |
|  | 2 | I'm not using them. | |
| Q15. Do you know about generic drugs? | | | |
|  | 1 | I know about them. | |
|  | 2 | I don’t know about them. | |
| Q16. (This question is only for those who answered "I know about them" in Q15.)  Please select all you know about generic drugs. | | | |
|  | 1 | The drug cost is lower compared with branded drugs. | |
|  | 2 | Efficacy is equivalent to branded drugs. | |
|  | 3 | Drugs manufactured and sold by another manufacturer after the branded drugs' patent has expired. | |
|  | 4 | Drugs approved by the country (Ministry of Health, Labour and Welfare) | |
|  | 5 | Medical expenses (pharmaceutical expenses) can be saved, helping to maintain the universal health insurance system. | |
|  | 6 | Some drugs do not have generic drugs. | |
|  | 7 | Equivalent quality, efficacy and safety to branded drugs. | |
|  | 8 | Excipients may differ from branded drugs. | |
|  | 9 | Depending on the drugs, there are multiple generic drugs for one brand name drug. | |
|  | 10 | Because of advances in formulation technology and ingenuity in formulation development by manufacturers, some have been improved in terms of color, shape, taste, etc. to make them easier to take. | |
|  | 11 | In some cases, the manufacture and sale of branded drugs has ended, and only generic drugs are available. | |
| Q17. Do you know authorized generic (AG) drugs? | | | |
|  | 1 | I know about them. | |
|  | 2 | I don’t know about them. | |
| Q18. (This question is only for those who answered "I know about them" in Q17.)  Do you know that AG drugs are generic drugs manufactured under license from the branded drug manufacturer? | | | |
|  | 1 | I know this. | |
|  | 2 | I don’t know this. | |
| Q19. (This question is only for those who answered "I know about them" in Q17.)  Do you know that AG drugs are basically the same as branded drugs except for the name, appearance and packaging?  (The active ingredient, drug substance, excipients, manufacturing method, manufacturing technology, etc. are all the same as the branded drug. *The manufacturing plant may differ.) | | | |
|  | 1 | I know this. | |
|  | 2 | I don’t know this. | |
| Q20. This question is only for those who answered "I know about them" in Q17.)  Regarding AG drugs, do you know that generic drugs, including AG drugs, are less expensive than branded drugs due to less development costs and time? | | | |
|  | 1 | I know this. | |
|  | 2 | I don’t know this. | |
| Q21. HLS-EU-Q47 | | | |
| Q22. EQ-5D-5L | | | |
| Q23. PDAS | | | |
| Q24. WPAI | | | |
| Q25. MIDAS | | | |

**Supplemental Table 5.** Reasons for healthcare visits in patients with treated chronic pain

|  | | **Treated Chronic Pain**  **n = 484**  **n (%)** | |
| --- | --- | --- | --- |
| Reasons for visiting healthcare institution in currently treated patients | Multiple responses allowed | Reaching the institution seems easy | 213 (44.0) |
|  |  | Anticipated treatment effect | 191 (39.5) |
|  |  | Superior expertise | 146 (30.2) |
|  |  | Healthcare professionals were kind and nice during my consultation | 122 (25.2) |
|  |  | Treatment cost seems low | 60 (12.4) |
|  |  | Waiting time seems short | 60 (12.4) |
|  |  | Other | 65 (13.4) |
|  | Primary Reason | Reaching the institution seems easy | 139 (28.7) |
|  |  | Anticipated treatment effect | 132 (27.3) |
|  |  | Superior expertise | 79 (16.3) |
|  |  | Healthcare professionals were kind and nice during my consultation | 43 (8.9) |
|  |  | Waiting time seems short | 19 (3.9) |
|  |  | Treatment cost seems low | 18 (3.7) |
|  |  | Other | 54 (11.2) |

**Supplemental Table 6.** Awareness of generic drugs and their relationship with healthcare visits in patients with chronic pain and migraines

|  | | **Chronic Pain** | | **Migraine** | |
| --- | --- | --- | --- | --- | --- |
|  | | **Untreated (%)**  **n = 605** | **Treated (%)**  **n = 484** | **Untreated (%)**  **n = 695** | **Treated (%)**  **n = 237** |
| Generic drug awareness questions | Aware | 579 (95.7) | 475 (98.1) | 591 (85.0) | 216 (91.1) |
| Question 1: The price is lower than brand-name drugs. | Yes | 525 (90.7) | 446 (93.9) | 522 (88.3) | 181 (83.8) |
| Question 2: The effectiveness is equivalent to brand-name drugs. | Yes | 393 (67.9) | 335 (70.5) | 368 (62.3) | 124 (57.4) |
| Question 3: Generic drugs are those sold by other manufacturers after expiration of the brand-name drug's patent. | Yes | 297 (51.3) | 273 (57.5) | 252 (42.6) | 107 (49.5) |
| Question 4: Generic drugs have been nationally approved (by the Ministry of Health, Labour and Welfare). | Yes | 232 (40.1) | 223 (46.9) | 217 (36.7) | 86 (39.8) |
| Question 5: Generic drugs are useful to maintain the system of public health insurance for the whole nation by saving costs (drug cost). | Yes | 182 (31.4) | 199 (41.9) | 165 (27.9) | 75 (34.7) |
| Question 6: Drugs without generic equivalents exist. | Yes | 241 (41.6) | 260 (54.7) | 245 (41.5) | 97 (44.9) |
| Question 7: The quality, efficacy and safety are equivalent to the brand-name drug. | Yes | 202 (34.9) | 194 (40.8) | 164 (27.7) | 66 (30.6) |
| Question 8: Additives may differ from the brand-name drug. | Yes | 123 (21.2) | 135 (28.4) | 122 (20.6) | 54 (25.0) |
| Question 9: Depending on the drug, multiple generic drugs may exist for one brand-name drug. | Yes | 158 (27.3) | 159 (33.5) | 148 (25.0) | 72 (33.3) |
| Question 10: Generic drugs with improved color, shape, and taste to make them easy to ingest may exist for which advanced formulation technology and ingenuity to develop new formulations were applied by manufacturers. | Yes | 105 (18.1) | 131 (27.6) | 103 (17.4) | 50 (23.1) |
| Question 11: There are drugs for which only generic drugs exist due to discontinued manufacturing and sale of the brand-name drug. | Yes | 76 (13.1) | 83 (17.5) | 83 (14.0) | 40 (18.5) |

**Supplemental Table 7.** Awareness of authorized generic drugs and their relationship with healthcare visits in patients with chronic pain and migraines

|  | | **Chronic Pain** | | **Migraine** | |
| --- | --- | --- | --- | --- | --- |
|  | | **Untreated (%)**  **n = 605** | **Treated (%)**  **n = 484** | **Untreated (%)**  **n = 695** | **Treated (%)**  **n = 237** |
| Authorized generic drug awareness questions | Aware | 49 (8.1) | 74 (15.3) | 99 (14.2) | 85 (35.9) |
| Question 1: Generic drugs are those that have been manufactured with authorization from the manufacturer of the brand-name drug. | Yes | 46 (93.9) | 65 (87.8) | 83 (83.8) | 78 (91.8) |
| Question 2: Except for the brand name and appearance/package, AG drugs are fundamentally the same as brand-name drugs (Note: for those drugs where the active component, raw ingredients, additives, production techniques, manufacturing factory, and manufacture technologies are the same as those of brand-name drugs, the manufacturing factory etc. may be different in certain cases). | Yes | 43 (87.8) | 61 (82.4) | 79 (79.8) | 73 (85.9) |
| Question 3: Prices of generic drugs, including AG drugs, are lower than the brand-name drugs due to the reduced cost and time related to their development. | Yes | 42 (85.7) | 64 (86.5) | 79 (79.8) | 70 (82.4) |

AG, authorized generic drug

**Supplemental Table 8.** Reasons for healthcare visits in patients with treated migraines

|  | | | **Treated Migraine**  **n = 237**  **n (%)** |
| --- | --- | --- | --- |
| Reasons for visiting healthcare institution in currently treated patients | Multiple responses allowed | Anticipated treatment effect | 89 (37.6) |
|  |  | Reaching the institution seems easy | 88 (37.1) |
|  |  | Healthcare professionals were kind and nice during my consultation | 71 (30.0) |
|  |  | Superior expertise | 65 (27.4) |
|  |  | Treatment cost seems low | 58 (24.5) |
|  |  | Waiting time seems short | 40 (16.9) |
|  |  | Other | 23 (9.7) |
|  | Primary Reason | Anticipated treatment effect | 56 (23.6) |
|  |  | Reaching the institution seems easy | 47 (19.8) |
|  |  | Superior expertise | 38 (16.0) |
|  |  | Treatment cost seems low | 27 (11.4) |
|  |  | Healthcare professionals were kind and nice during my consultation | 26 (11.0) |
|  |  | Waiting time seems short | 23 (9.7) |
|  |  | Other | 20 (8.4) |
